# Natural killer cells in sepsis: Friends or foes?

**DOI:** 10.3389/fimmu.2023.1101918

**Published:** 2023-01-26

**Authors:** Fangjie Wang, Yiqin Cui, Dongmei He, Lisha Gong, Huaping Liang

**Affiliations:** ^1^ State Key Laboratory of Trauma, Burns and Combines Injury, Department of Wound Infection and Drug, Daping Hospital, Army Medical University (Third Military Medical University), Chongqing, China; ^2^ School of Laboratory Medicine and Technology, Harbin Medical University, Daqing, China

**Keywords:** natural killer cells, sepsis, immunotherapy, protective effect, detrimental effect

## Abstract

Sepsis is one of the major causes of death in the hospital worldwide. The pathology of sepsis is tightly associated with dysregulation of innate immune responses. The contribution of macrophages, neutrophils, and dendritic cells to sepsis is well documented, whereas the role of natural killer (NK) cells, which are critical innate lymphoid lineage cells, remains unclear. In some studies, the activation of NK cells has been reported as a risk factor leading to severe organ damage or death. In sharp contrast, some other studies revealed that triggering NK cell activity contributes to alleviating sepsis. In all, although there are several reports on NK cells in sepsis, whether they exert detrimental or protective effects remains unclear. Here, we will review the available experimental and clinical studies about the opposing roles of NK cells in sepsis, and we will discuss the prospects for NK cell-based immunotherapeutic strategies for sepsis.

## 1 Introduction

Sepsis is a life-threatening multiple-organ dysfunction syndrome caused by localized or systemic infections, which is one of the major causes of death to patients in the hospital worldwide (1–3). It has been estimated that approximately 750,000 people suffer from sepsis every year in the United States and an estimated 20-30% patients die from it (4, 5). However, there is no specific, standardized treatment strategy for sepsis (6). Numerous studies have shown that dysregulation of innate immune responses is a major contributing factor to the incidence and development of sepsis (7, 8). For example, studies on monocytes, macrophages, neutrophils, and dendritic cells have provided insight into their roles in both the inflammatory and immunosuppressive phases of sepsis (9–14). Natural killer (NK) cells, which were discovered in the early 1970’s (15, 16), are a heterogeneous group of innate lymphocytes with the capacity to regulate both innate and adaptive immune responses. They are best known for their roles in fighting infections and tumors, mainly relying on their cytotoxicity and immune regulatory properties (17).

Recent studies have implicated NK cells in the pathological process of sepsis, suggesting that they might be employed as prognostic biomarkers or therapeutic targets (2, 18). However, seemingly contradictory conclusions about NK cells playing beneficial or harmful roles in sepsis have been obtained (19). Hence, we will review these reports to discuss whether NK cells are friends or foes in sepsis, and we will further discuss the prospects of NK cell-based immunotherapy for sepsis.

## 2 The immunological characteristics of sepsis

**Figure 1:**
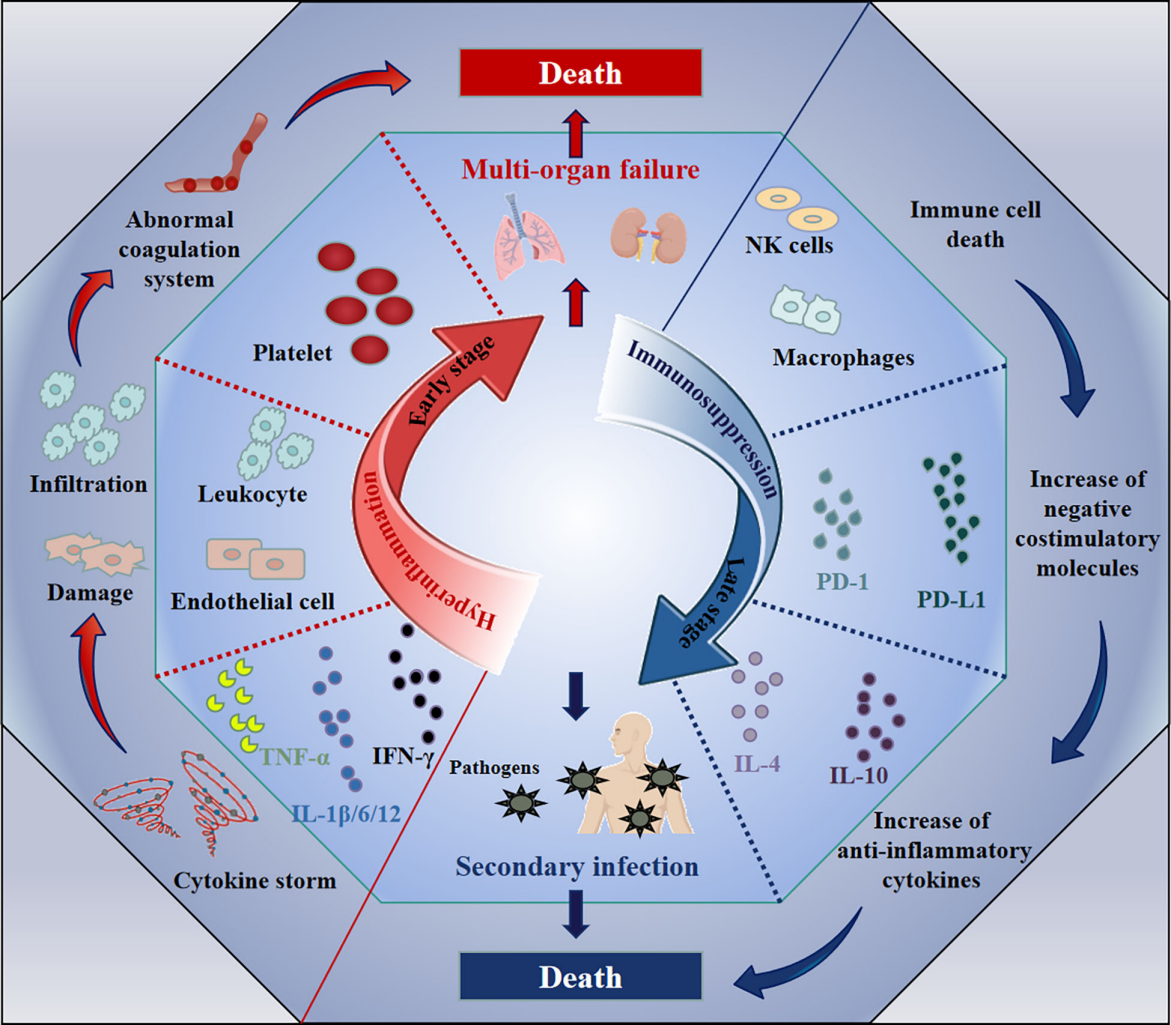
The immune changes during the pathological process of sepsis. The immunological abnormity exhibits two distinct stages accompanying with the sepsis development: hyperinflammation and immunosuppression. During the hyperinflammatory phase at early, the increase of pro-inflammatory cytokines (e.g., IL-1β/6/12, IFN-γ, and TNF-α) leads to cytokine storm, resulting in the vascular system damage (e.g., endothelial cell damage), the abnormal coagulation, finally multi-organ failure and death. Subsequently, the death of immune cells, the increase of negative costimulatory molecules (e.g., PD-1 and PD-L1) or anti-inflammatory cytokines (e.g., IL-4 and IL-10) induces immunosuppression, which leads to uncontrolled secondary infection and death.

Sepsis has previously been used to describe severe disease caused by infection (20). However, this definition cannot accurately describe its complex pathological processes. Recently, a new definition has been published, stating that sepsis refers to a life-threatening, multiple-organ failure syndrome, caused by dysregulated responses to infection (21, 22). It is generally believed that immunological abnormalities are the pathological basis of sepsis (23), which is tightly associated with microvascular injury, abnormal coagulation, hemodynamic instability, multiple organ damage and other conditions (24). The immunological abnormities exhibit distinct disease stage-specific characteristics during sepsis: hyperinflammation at the initial stage and immunosuppression at the late stage (25). A diagram illustrating this process is shown in **Figure 1**.

After invading the body, pathogens will encounter the first line of defense composed of innate immune cells, activating PAMP (pathogen-associated molecular pattern)- or DAMP (damage-associated molecular pattern)-associated signaling pathways in these cells (26, 27). Once activated, these cells generate large amounts of inflammatory cytokines, such as IL-1β, IL-6, IL-12, TNF-α and IFN-γ (28, 29). These host responses are not limited to the infectious focus. The cytokines may trigger additional immune cells at distant sites to secrete inflammatory cytokines, and this cascading amplification reaction may finally result in systemically uncontrolled over-inflammation, which is termed a “cytokine storm” (30, 31). The massively increased cytokine levels may potentially enhance the elimination of pathogens by innate immune cells. However, they also lead to a series of pathological changes, such as endothelial cell damage, leukocyte infiltration, abnormal activation of the coagulation system and other abnormalities, resulting in multi-organ failure and even death (32–34). Consequently, the direct cause of death is not the invasive pathogens themselves, but the over-activated immune reactions. Therefore, the focus of clinical treatment at this inflammatory stage of sepsis is on ameliorating the uncontrolled inflammation (35).

The hyperinflammation at the early stage of sepsis will lead to immunosuppression during the late stage of sepsis: on the one hand, the cytokine storm directly induces cell death in various immune cells; on the other hand, the functions of some effector cells will be exhausted after their excessive activation (36, 37). Moreover, upregulation of some negative costimulatory molecules and anti-inflammatory cytokines has also been observed during this stage, and includes programmed cell death 1 (PD-1) (38), programmed cell death ligand 1 (PD-L1) (39), T-cell immunoglobulin and mucin domain-containing protein-3 (TIM-3) (40), T cell Ig and ITIM domain (TIGIT) (41), IL-4 (36), IL-10 (42, 43) and TGF-β (44, 45). These factors are mainly related to exhaustion of immune cells or inhibition of their effector functions (38, 46–48). As a result, the body presents with a continuously immunosuppressive state, nearly losing its capacity to clear pathogens (49). This will cause an extremely high risk for secondary infections, such as those mediated by opportunistic pathogens or iatrogenic infections caused by interventional therapy, which eventually leads to death of sepsis patients (50). For example, Huang et al. observed that the expression of TIM-3 on CD4 T cells in patients with sepsis-induced immunosuppression was significantly elevated, which impaired anti-infective responses and positively correlated with mortality (51). Hou et al. also found that, in a lipopolysaccharide (LPS)-induced murine sepsis model, TIM-3 expression on NK cells negatively regulated the production of IFN-γ, which caused death (40). Therefore, reestablishing immune functions is critical to reduce mortality risk of sepsis patients during the late immunosuppressive stage (52, 53).

## 3 NK cells play a role in antimicrobial responses

NK cells, a group of large granular lymphocytes derived from the bone marrow, are essential components of the innate immune response and can directly kill tumors and other target cells without prior activation (54–56). In humans, about 5-15% of lymphocytes are defined as NK cells in peripheral blood, and tissue-specific subpopulations are found in the spleen, liver, and lung (57–61). Generally, human NK cells can be divided into two subpopulations by the expression of CD56 and CD16 on the cell membrane (62, 63). About 90% of all NK cells in human peripheral blood are CD56^dim^CD16^bright^, whereas only 10% are CD56^bright^CD16^-/dim^ (64). Distinct human NK cell subpopulations found in different tissues significantly differ in cytotoxicity and cytokine secreting capacity (65, 66). The two main subpopulations possess distinct functions: CD56^dim^CD16^bright^ NK cells exhibit higher cytotoxicity and express increased levels of killer immunoglobulin-like receptors (KIR) or CD57 receptors; CD56^bright^CD16^-/dim^ NK cells can secrete more cytokines and possess greater proliferative capacity (67, 68).

NK cells can be activated in several ways. Most importantly, the balance between signals from the inhibitory or activating receptors expressed on the cell surface plays a critical role in regulating their responses (69, 70). The activating receptors mainly include NCRs (NKp30, NKp44, and NKp46), KIR-2Ds, KIR-3Ds, NKG2D, CD226, 2B4, and NKG2C, whereas the inhibitory receptors mainly include NKG2A, TIGIT, KIR-2DL, and KIR-3DL (71). The biased expression of these receptors or their ligands calibrates the activation status of NK cells. For example, a clinical study reported that, in human immunodeficiency virus (HIV)-infected patients, a subpopulation of human NK cells that expresses NKG2C but not NKG2A has a stronger ability to secrete IFN-γ compared with other NK cells (72). Another typical way of NK activation is *via* their pathogen recognition receptors (PRRs), which bind with PAMPs on bacteria (73). For example, a previous study reported that high-mobility group box-1 (HMGB-1) up-regulated the levels of TLR-2/4, which belongs to the group of classical PRRs (74), on murine NK cells, leading to their activation in rotavirus-induced murine biliary atresia (75). Additionally, NK cells can also be activated by several cytokines, including type 1 interferon, IL-2, IL-12, IL-15, IL-18, IL-21, and IL-27 (76–80). For instance, IL-12 binding to IL-12Rβ1/2 stimulates NK cells through signal transducer and activator of transcription 4 (STAT4) phosphorylation, leading to abundant IFN-γ and TNF-α production (81).

During infection, activated NK cells perform their activity mainly in two ways: cytotoxicity and immune regulation. First, NK cells can directly lyse bacteria-infected cells with their cytotoxicity: on the one hand, they can induce target cell apoptosis depending on the binding of FAS-L to FAS death receptors (82); on the other hand, they directly kill targets by secreting cytotoxic proteins, such as perforin, granzyme and α-defensins (83–85). Specifically, some studies have reported that these cytotoxic proteins could disrupt the membrane of some bacteria, such as *Mycobacterium, Salmonella typhimurium*, *Bacillus anthracis*, *Escherichia coli*, and *Staphylococcus aureus (* 86–89), thus causing their death. In addition to cytotoxicity, activated NK cells also secrete several cytokines to undertake the roles of immune regulation (90). IFN-γ, which is the major cytokine released by NK cells, was reported to play a critical role in fighting microbial infections (91). It modulates the activation of other immune cells, such as macrophages or dendritic cells, enabling them to perform comprehensive anti-bacterial responses (92, 93). Moreover, IL-32, previously named as NK cell transcript 4 (NK4), can be produced by NK cells when activated by IL-2 (94, 95). It also stimulates inflammatory responses by inducing monocytes or macrophages to secrete various cytokines, including TNF-α, IL-1β, IL-6 or IL-8 (96). Thus, IL-32 has been reported to exacerbate sepsis in the cecal-ligation and puncture (CLP) mouse model, *via* propagating vascular inflammation (97).

In addition to their positive regulatory roles, NK cells also possess the ability to limit antimicrobial responses. A recent study uncovered that NK cell-derived IFN-γ worsened macrophage phagocytosis of zymosan in mice and increased the susceptibility to secondary *Candida* infection during post-sepsis immunosuppression (98). However, whether this phenomenon exists in sepsis caused by other pathogens needs further study. Furthermore, activated NK cells also secrete IL-10, which is a well-known immunosuppressive cytokine (99–101). In fact, NK cells are the main source of IL-10 in systemic infection caused by some pathogens, such as *Yersinia pestis*, *Listeria monocytogenes* or *Toxoplasma gondii (* 99). Interestingly, the NK cell-derived IL-10 appears to play dual roles in different types of infections. For example, in *Listeria monocytogenes* infection, the NK cell-derived IL-10 shows detrimental effects on host resistance against the invasive pathogen (102), whereas it can protect the host from murine cytomegalovirus infection or CLP-induced sepsis by reducing systemic inflammation (103, 104). The authors consider that the beneficial or detrimental roles of IL-10 might depend on whether the major cause of host death is pathogen overload or excessive inflammation during infection.

**Figure 2:**
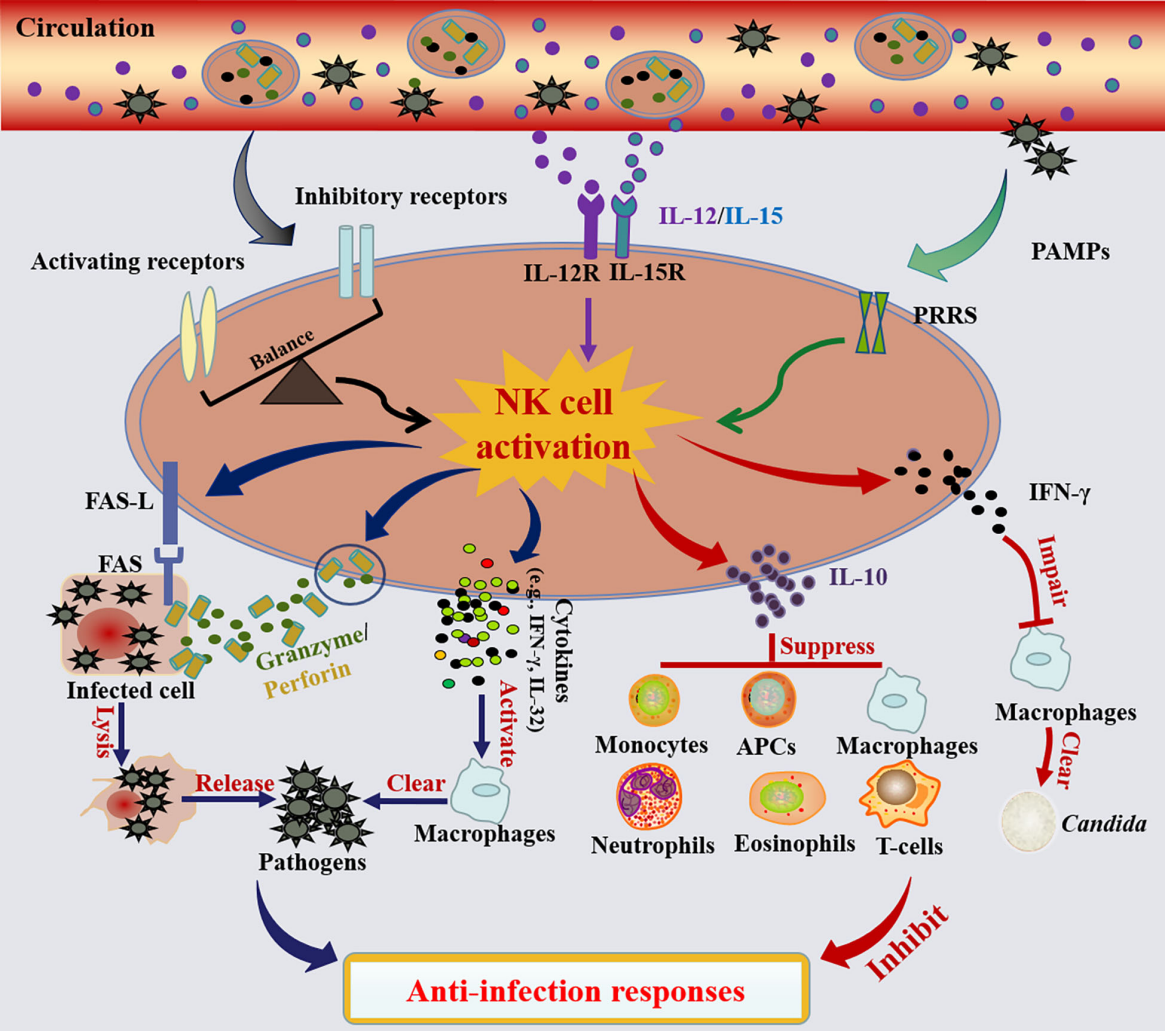
NK cell activation and their roles in the anti-infection responses. NK cells are mainly activated in three ways: 1) The activation of NK cells is governed by a balance between signals delivered through activated and inhibitory receptors. When the activating signal dominates, NK cells will be activated, and vice versa. 2) Activation of NK cells can also be achieved by stimulation with cytokines (e.g., IL-12 and IL-15). 3) NK cells are activated by pathogen-associated molecular patterns (PAMPs) through pattern recognition receptors (PRRs). Activated NK cells lysis infected cells and release pathogens *via* death receptor ligand/death receptor (e.g., FAS-L/FAS) and secreting cytotoxic proteins (e.g., perforin and granzyme). Meanwhile, activated NK cells promote the activation of macrophage-mediated microbial killing by the secretion of cytokines (e.g., IFN-γ, IL-32). In contrast, activated NK cells also possess the ability to limit the anti-infection responses. On one hand, NK cell-derived IFN-γ especially worsened macrophage phagocytosis of zymosan.; on the other hand, the activated NK cells also secrete IL-10, which can generally inhibit the anti-infection responses of monocytes, antigen-presenting cells (APCs), macrophages, neutrophils, eosinophils or T cells.

Summarily, the patterns of NK cell activation and their roles in antimicrobial responses are illustrated in **Figure 2**.

## 4 NK cells act as risk factors in sepsis

**Figure 3:**
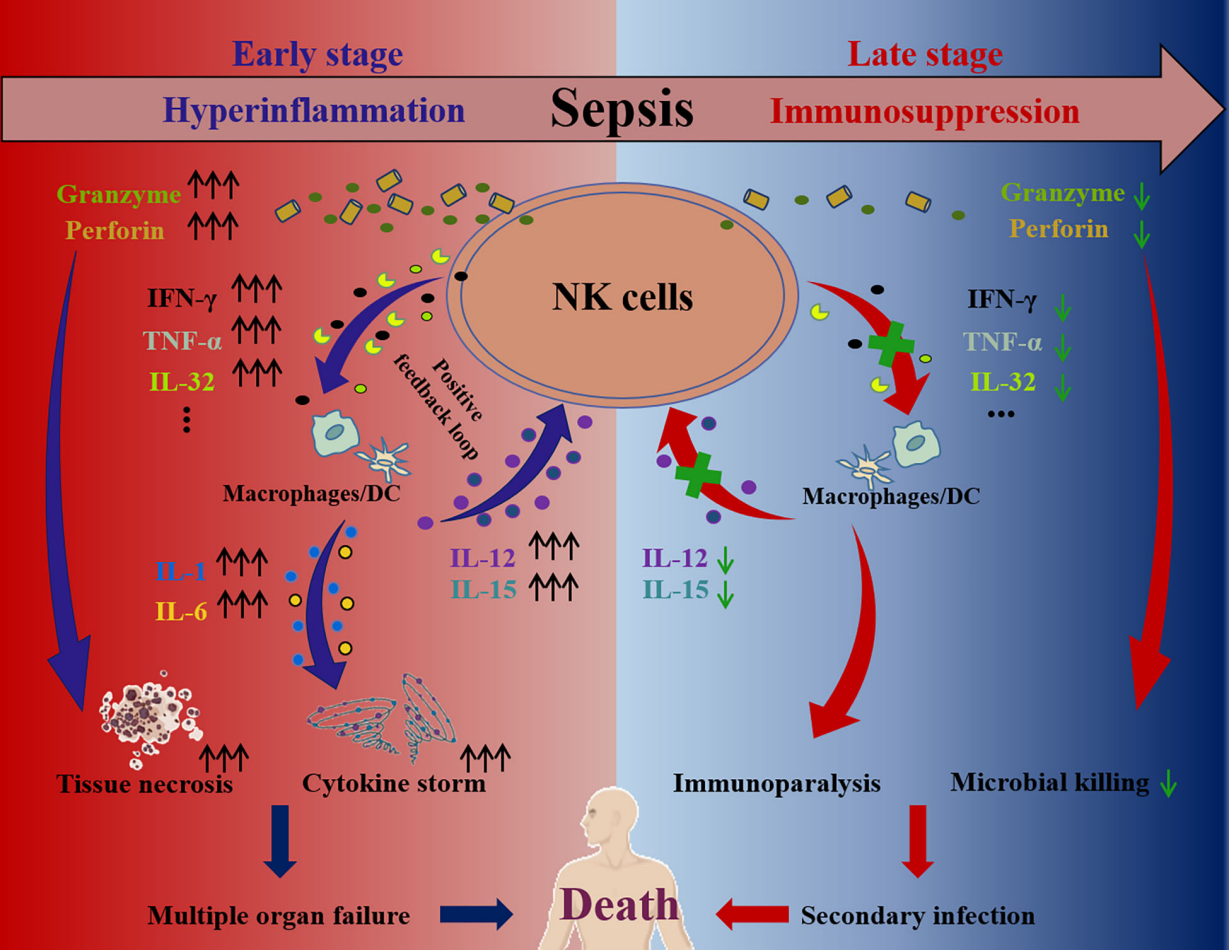
The pathological roles of NK cell at the hyperinflammation and immunosuppression stage of sepsis. During sepsis hyperinflammation, NK cells activation is dysregulated and NK cells secrete abundant cytokines, including IFN-γ, TNF-α, IL-32 and so on. These cytokines subsequently facilitate secretion of more cytokines (e.g., IL-12, IL-15, IL-1, IL-6, and so on) by dendritic cells and macrophages, establishing a positive feedback loop and amplifying cytokine storm. Furthermore, cytotoxic proteins (e.g., perforin, granzyme) secreted by NK cells are increased and cause tissue necrosis. As a result, the cytokine storm and tissue necrosis eventually lead to multiple organ failure and death. In contrast, the secretions of cytokines (e.g., IFN-γ, TNF-α, IL-32, and so on) and cytotoxic proteins (e.g., perforin, granzyme) of NK cells are impaired at the immunosuppression stage of sepsis, which contributes to the immunoparalysis, causing secondary infection and even death.

Accumulating studies have shown that NK cells play a contributing role in the inflammatory responses caused by infection (105, 106). In this context, they are considered a risk factor for aggravating the septic process during the hyperinflammation stage (107). At the early stage of sepsis, NK cells will be activated through the ways discussed above, secreting abundant cytokines, such as IFN-γ, TNF-α or IL-32, which can trigger dramatic responses in macrophages or dendritic cells (54, 96). Mutually, the activated macrophages and dendritic cells secrete IL-2, IL-12 or IL-18 to subsequently further activate NK cells, forming a positive feedback loop (108, 109). This loop amplifies the pro-inflammatory responses, resulting in a cytokine storm and finally causing multiple organ failure (54). In addition, the cytotoxic proteins secreted from activated NK cells, including perforin and granzyme, are also reported to directly mediate tissue necrosis and damage (54) (**Figure 3**). Therefore, several studies have shown that antagonizing murine NK cells during sepsis significantly ameliorates multiorgan damage caused by inflammation and enhanced tolerance in mice. For example, in sepsis mouse models caused by CLP surgery, *Streptococcus pneumoniae*, *Escherichia coli* or *Streptococcus pyogenes* infection, NK cell clearance using anti-asialoGM1 and anti-NK1.1 antibodies can reduce systemic inflammation, stabilize acid-base balance in the circulation, improve organ damage, reverse physiological disorders and prolong overall survival (110–116). Moreover, in a murine polytrauma model, which is a major instigator of sepsis, murine NK cell depletion also attenuated inflammatory responses and improved the outcomes (117).

IL-15 is an essential cytokine to maintain NK cell development and maturation, which can also strongly activate NK cells at high concentrations (118). It has been reported that excessive IL-15 stimulation leads to pathological inflammatory responses similar to sepsis, resulting in the death of mice due to massive NK cell proliferation and IFN-γ production (119). Furthermore, IL-15 knockout mice, characterized by NK cell loss, also showed tolerance to sepsis due to CLP surgery (120). When bacterial infection occurs, NK cells may rapidly migrate to the infection site and promote inflammation (121, 122). It has been reported that murine NK cells expressing CXCR3 can rapidly migrate to the abdominal cavity within 4-6 h following severe abdominal infection (123). These CXCR3-positive NK cells are similar to the human CD56^bright^ subpopulation in their ability to secrete more proinflammatory cytokines and express more activation makers (124). Blocking CXCR3 or its ligand, CXCL10, can significantly reduce inflammation during sepsis in mice and increase their survival rate (125). In addition to the organ damage caused by massive inflammatory cytokine secretion, NK cell-mediated cytotoxicity is also detrimental in sepsis. For example, mice deficient in perforin or in granzymes A/M exhibit increased tolerance to sepsis caused by LPS (126).

Additionally, significant changes in the number, phenotypes, and functions of NK cells in sepsis patients have been observed in several clinical studies. David Andaluz-Ojeda et al. showed that NK cell levels were significantly increased in patients who died from sepsis and the cell counts at day 1 were independently associated with increased risk of death at 28 days (hazard ratio = 3.34, 95% CI = 1.29 to 8.64; *P* = 0.013). Analysis of survival curves provided evidence that human NK cell levels at day 1 (> 83 cells/mm³) were associated with early mortality (127). Palo et al. also found that sepsis patients with the highest NK cell numbers exhibit the lowest survival probability (128).

In all, during the hyperinflammation stage, the disturbance of inflammatory factors leads to abnormal NK cell activation, which can trigger a cytokine storm through a positive feedback loop, resulting in severe organ damage (92, 109). Thus, neutralizing or inhibiting NK cell-derived pro-inflammatory cytokines (e.g., IFN-γ) or cytotoxic proteins (e.g., perforin, granzyme) can alleviate systemic inflammatory responses and protect against organ damage. Furthermore, using anti-inflammatory cytokines, such as IL-10, to treat sepsis is also worth considering. We have summarized the evidence showing the detrimental roles of NK cells from both animal and human sepsis in **Table 1**. These findings implicate NK cells as risk factors during sepsis.

**Table 1:** Summary of the detrimental roles of NK cells in sepsis.

Disease	Animal/Human	Supporting evidence	Reference
CLP	Animal	Using anti-asialoGM1 and anti-NK1.1 antibodies to clear NK cells *in vivo* enhanced tolerance in mice	(110–113)
*E. coli* infection	Animal	NK cell-depleted and NK cell-deficient mice exhibited 80% survival after *E. coli* infection, whereas control mice all died within 12 h.	(114)
*S. pyogenes* infection	Animal	NK cell-deficient mutant mice were more resistant to *S. pyogenes* than control mice	(115)
*S. pneumonia* infection	Animal	NK depletion by antibodies reduced systemic inflammation, stabilized acid-base balance in circulation, and significantly improved the survival of mice	(116)
Murine polytrauma	Animal	Depleting NK cells resulted in attenuated inflammatory responses and an overall improvement in outcome	(117)
CLP	Animal	IL-15-deficient mice (lacking NK cells) exhibited improved survival, attenuated hypothermia, and reduced proinflammatory cytokine production during sepsis	(120)
Patients within the first 1 d, 3 d, 10 d of sepsis (50 patients)	Human	Analysis of survival curves provided evidence that NK cell levels at day 1 (> 83 cells/mm³) were associated with early mortality	(127)
Patients with sepsis during the first 28 d in the ICU (52 patients)	Human	Patients with the highest NK cell number may have the lowest probability to survive	(128)

CLP, Cecal-ligation and puncture; E. coli, Escherichia coli; S. pyogenes, Streptococcus pyogenes; S. pneumonia, Streptococcus pneumoniae.

## 5 The protective roles of NK cells in sepsis

Conversely, some other studies have provided evidence for a protective role of NK cells in a variety of microbial infections. For instance, murine NK cells are essential in coordinating host responses against sepsis caused by *Staphylococcus aureus* infection (129, 130). This may be due to their interactions with the anti-inflammatory mechanisms of the host. Moreover, once the ability of NK cells to secrete IFN-γ is impaired, progressive immune disorders might be induced. There is evidence showing that neutralization of IL-10 with antibodies in mice improves the ability of NK cells to secrete IFN-γ, resulting in improved survival (131). Notably, in the *Citrobacter rodentium* infection model, murine NK cells not only directly lyse the bacteria but also recruit other intrinsic immune cells and activate their antibacterial functions by secreting cytokines (132). Similarly, during *Pseudomonas aeruginosa* infection, NK cells can recruit neutrophils to the lungs, alleviating infection and improving animal survival (133). In mice infected with pulmonary nontuberculous mycobacteria, the bacterial load and mortality rate are increased by NK cell clearance (134). Interestingly, it has also been reported that IL-15 treatment after CLP surgery can reduce immune cell apoptosis, improve immune disorders, and increase mouse survival (135, 136).

A protective role of NK cells in sepsis has also been documented in several clinical studies. Some researchers reported a significant increase in the number of human peripheral blood NK cells, their expression of active biomarkers, and their ability to secrete granzyme A/B, IFN-γ or IL-12P40 (117, 137–139), which were considered to provide a survival benefit for septic patients. Bourboulis et al. showed that sepsis patients with increased levels of NK cells (>20% of all lymphocytes) survived longer than those patients with lower levels of NK cells (< or =20% of all lymphocytes) (140). Boomer et al. reported that NK cells in peripheral blood of sepsis patients were significantly reduced within 24 h, which may predispose some patients to nosocomial infections and poor outcomes (141). Consistently, Holub et al. found that human NK cells were decreased within the first 48 h of sepsis, especially in patients with Gram-negative bacterial infection, resulting in increased risk of septic complications (142). Moreover, single-cell RNA-sequencing (scRNA-seq) analysis revealed that various cytotoxic genes of NK cells were downregulated in patients with late sepsis (n=4), which might be associated with the re-occurrence of severe infections (143).

Under the conditions described in this section, replenishing subjects with functional NK cells may hinder the immunosuppressive stage of sepsis. Furthermore, blocking inhibitory receptors, activating NK cells by cytokines (e.g., IL-15, IL-2) or neutralizing suppressive cytokines (e.g., IL-4, IL-10) may also be beneficial. In summary, the evidence supporting the protective roles of NK cells in both animal and clinical studies are shown in **Table 2**.

**Table 2:** Summary of the protective roles of NK cells in sepsis.

Disease	Animal/Human	Supporting evidence	Reference
*S. aureus* infection	Animal	NK cell-depleted mice (using anti-NK1.1 antibodies) developed more frequent and severe arthritis	(129, 130)
*C. rodentium* infection	Animal	Depletion of NK cells led to higher bacterial load and developed disseminated systemic infection, associated with reduced immune cell recruitment and lower cytokines	(132)
*P. aeruginosa* infection	Animal	NK cells can recruit neutrophils to the lungs, alleviate infection and improve the survival of mice	(133)
NTM infection	Animal	NK1.1 cell depletion increased bacterial load and mortality in mouse model	(134)
Patients within 12 h of the advent of severe sepsis (49 patients)	Human	An increase in circulating NK cells increased the survival rate of patients	(140)
Patients within 24 h of the onset of sepsis (24 patients)	Human	The number of NK cells in the blood of patients was decreased, which may be necessary for predisposing some patients to nosocomial infection and poor outcome	(141)
Patients within 48 h of sepsis (40 patients)	Human	NK cells numbers steadily decreased within 48 hours after admission, associated with an increased risk of septic complications	(142)
Patients with sepsis during 14-21 d (4 patients)	Human	Various cytotoxic genes of NK cells were downregulated in patients with late sepsis, which might be associated with the re-occurrence of severe infections	(143)

S. aureus, Staphylococcus aureus; C. rodentium, Citrobacter rodentium; P. aeruginosa, Pseudomonas aeruginosa; NTM, Nontuberculous mycobacteria.-

Taken together, the roles of NK cells in sepsis remain controversial. Furthermore, animal and clinical studies have revealed dual roles of NK cell activity on sepsis progression. The impact on disease mainly depends on the pathological stage and the initial infection focus. Although the functional changes of NK cells and their influence on pathological progresses have been explored in previous studies, they mainly focused on the early stages after sepsis. During the sepsis process lasting several months from occurrence to recovery, the impact of continuous changes in NK cell numbers and characteristics remains unclear.

## 6 NK cells in COVID-19 infection

In late 2019, coronavirus disease 2019 (COVID-19) emerged and rapidly spread throughout the world (144, 145). As of December 2022, the COVID-19 pandemic has resulted in approximately 641,915,931 confirmed cases, including 6,622,760 deaths worldwide (https://covid19.who.int/ ). A meta-analysis revealed that the overall pooled sepsis prevalence estimates among 218,184 COVID-19 patients, irrespective of ICU or non-ICU admission, were 51.6% (95% CI, 47.6-55.5, *I* ^2^ = 100%) (146). Sepsis was one of the major causes of death for COVID-19 patients. During acute COVID-19 infection, the number of the CD56^bright^ and CD56^dim^ human NK cells dropped dramatically in the circulation (147, 148). However, this drop was likely related to the homing of human NK cells from the circulation to the lung because NK cells were increased in bronchoalveolar lavage (BAL) (149, 150). Moreover, a clinical trial discovered that a high frequency of NK cells was significantly associated with asymptomatic COVID-19 infection (151). In addition to lower circulating counts, NK cell dysfunction was also observed. NK cell hyperactivation driven by IL-6, IL-15 and IL-18 has been considered as one of the features of COVID-19 (152–154). Furthermore, Maucourant et al. used high-dimensional flow cytometry to reveal that NK cells in COVID-19 patients were at a higher activation state containing high levels of cytotoxic proteins, such as perforin (155). However, prolonged hyperactivation usually leads to impaired NK cell function. Yao et al. reported that genes involved in NK cell cytotoxicity were suppressed in severely ill COVID-19 patients (156). Moreover, some studies also reported that NK cell activity was impaired *via* over expression of the inhibitory receptor NKG2A in COVID-19 patients (157, 158).

Due to their lower circulating counts and dysfunction, NK cell adoptive transfer or reconstitution could be a possible treatment for COVID-19 patients. In fact, some innovate clinical trials using human NK cells to treat COVID-19 patients are active (ClinicalTrials.gov# NCT04280224, NCT04578210). Additionally, a clinical trial to determine the safety and efficacy of NK cells derived from human placental hematopoietic stem cells in patients with moderate COVID-19 is also ongoing (ClinicalTrials.gov# NCT04365101). Finally, an NKG2D chimeric antigen receptor (CAR)-NK cell-based trial may provide a safe and effective cell therapy for COVID-19 (ClinicalTrials.gov# NCT04324996). These studies are summarized in **Table 3**.

**Table 3:** Summary of the clinical trials on NK cell-based immunotherapy.

Disease type	Patient number		Cell source	Supporting evidence	Phase		Reference or identifier
COVID-19	30		−	−	I (recruiting)		NCT04280224
COVID-19	58		Allogeneic	−	I/II (recruiting)		NCT04578210
COVID-19	86		Human placental hematopoietic stem cell	−	I/II (Active, not recruiting)		NCT04365101
COVID-19	90		CAR	−	I/II (recruiting)		NCT04324996
AML	21		Haploidentical	All patients but 1 had absolute neutrophil and platelet count recovery within 45 d after NK cell infusion	II (completed)		(159)
AML	10		UCB	*In vivo*, hematopoietic stem and progenitor cell-NK cell maturation was observed, indicated by the rapid acquisition of CD16 and most activating receptors	−		(160)
NHL	16		Haploidentical	Three responding patients with extensive bulky disease had robust tumor regressions	II (completed)		(161)
Neuroblastoma	35		Haploidentical	Ten of thirty-five patients had complete or partial responses and had improved progression free survival	I (completed)		(162)
MM	8		Allogeneic	After fresh NK cell infusion, dramatic *in vivo* expansion was observed and circulating NK cells retained the ability to kill myeloma cells	−		(163)
NHL and CLL	11		CAR	8 patients had an objective response, including 7 patients who had a complete response	I/II (Active, not recruiting)		(164)
Ovarian carcinomas	12		UCB	−	I (recruiting)		NCT03539406
Hematological cancer	37		iPSCs	−	I (Active, not recruiting)		NCT03841110
B cell lymphoma	234		iPSCs	−	I (recruiting)		NCT04023071
Glioblastoma	42		CAR	−	I (recruiting)		NCT03383978
HIV	9		Haploidentical	−	I (completed)		NCT03899480
HIV	4		Haploidentical	−	I (completed)		NCT03346499

Identifier from ClinicalTrials.gov. COVID-19, Coronavirus disease 2019; AML, Acute myeloid leukemia; NHL, Non-Hodgkin lymphoma; MM, Multiple myeloma; CLL, Chronic lymphocytic leukemia; HIV, Human immunodeficiency virus; CAR, Chimeric antigen receptors; UCB, Umbilical cord blood; iPSCs, Induced pluripotent stem cells.

## 7 The prospects of NK cell-based immunotherapy for sepsis

Recently, NK cells have gained great attention in the field of immunotherapy, especially in cancer treatment. The anti-tumor activities of infused NK cells have been demonstrated widely in mouse models of glioblastoma, ovarian cancer, and metastatic colorectal cancer (165–167). For example, Veluchamy et al. showed that adoptive transfer of NK cells into mice with metastatic colorectal cancer inhibited tumor growth *in vivo* and prolonged survival time (168). There has an explosion of NK cell-based cancer immunotherapies in clinical trials on acute myeloid leukemia (AML), non-Hodgkin lymphoma (NHL), neuroblastoma, multiple myeloma (MM) and other cancers (159–164). In addition, a few clinical trials using NK cells to treat patients with ovarian carcinomas, hematological cancer, B cell lymphoma, and glioblastoma are ongoing (ClinicalTrials.gov# NCT03539406, NCT03841110, NCT04023071, NCT03383978). We have summarized these completed and ongoing clinical trials in **Table 3**. Recently, a variety of NK cell-based immunotherapies were developed to treat viral infections such as COVID-19 (as discussed above) and HIV (ClinicalTrials.gov# NCT03899480, NCT03346499). Although these treatments have not yet achieved the same degree of success as clinical T cell-based therapies, the abundant pre-clinical or clinical studies with NK cell-based immunotherapies have led to increasing enthusiasm in exploring their potential to treat other diseases, including sepsis.

A variety of tissue sources for deriving NK cells for immunotherapy have been developed, including autologous and allogeneic NK cells (169). Autologous NK cell infusion using the patient′s own blood as a source was the first focus in adoptive NK cell therapy, which is associated with low risk of graft-versus-host disease (169). However, this approach usually leads to exhausted NK cell functions (170). Furthermore, patients must receive an extensive preparative treatment regimen before infusion, which may cause serious negative side effects (171). For allogeneic NK cells, the requirement for a healthy donor as source of NK cells and expanding them to clinically relevant doses is the most critical step (172). Therefore, umbilical cord blood (UCB) (173) and induced pluripotent stem cells (iPSCs) have been considered as optimal sources (174). UCB NK cells are younger and more proliferative (175), can be manufactured at multiple doses (176), and possess high cytotoxicity to lyse target cells (177). However, UCB NK cells are relatively unstable due to common delays in blood collection and heterogeneity of leukocytes from different donors (169). Stem cells represent a potentially unlimited source of NK cells for adoptive immunotherapy, and iPSCs provide a universal cell source (174). NK cells derived from iPSCs can be genetically modified and expanded to a homogenous population on a large scale (178). Furthermore, NK cells derived from iPSCs display increased cytotoxicity and greater antitumor activity than UCB NK cells in models of leukemia (179). However, more efficient strategies to generate NK cells from iPSCs are still needed.

As discussed above, NK cells significantly impact the pathological progression of sepsis. We postulate that NK cell-based immunotherapies may be developed as an excellent therapeutic option for sepsis, for the following reasons: 1. The adoptive transfer of NK cells has been proven safe due to their short lifespan and the low risk of triggering graft-versus-host reactions (180, 181); 2. NK cells can kill targets without sensitization; therefore, developing NK cells as “off-the-shelf” products has recently attracted great attention in the field (182), which can overcome the challenging problem of the narrow time window available for sepsis treatment; 3. The pathological process of sepsis is characterized by distinct stages of hyperinflammation and immunosuppression, and NK cells also have dual roles in immune regulation. Therefore, we may envisage an “off-the-shelf” NK cell product developed from editable iPSC-NK cells, which can sense its immune microenvironment to program opposing activities: in a hyperinflammatory environment, these NK cells may be programmed to mainly exert anti-inflammatory properties, whereas in an immunosuppressive environment, they are programmed to promote immune activation. Although few studies on NK cell-based immunotherapies for sepsis have been performed, inspired by explorations on cancer and viral infection and with the expanded knowledge on mechanisms of NK cell responses in sepsis, we can make the bold prediction that the future of NK cell-based immunotherapy for sepsis is bright.

In conclusion, developing NK cell-targeted immunotherapeutic strategies for sepsis highly depends on the disease state. A dynamic and more comprehensive understanding of the pathological process of sepsis will be critically important. Therefore, we consider using high-throughput sequencing technologies to dynamically monitor NK cell alterations during the early, middle, and late stages of sepsis essential for an accurate and deep understanding of NK cells in sepsis. Hopefully, with the growing understanding about NK cells in sepsis, safer and more efficient immunotherapies for sepsis can be developed.

## Author contributions

The work presented was performed in collaboration by all authors. FW and YC designed and wrote the manuscript. DH revised the manuscript. LG improved the language. HL devised the concept and revised the paper. All authors contributed to the article and approved the submitted version.
